# Paclitaxel Induced MDS and AML: A Case Report and Literature Review

**DOI:** 10.1155/2016/8308179

**Published:** 2016-02-29

**Authors:** Udit Bhaskar Bhatnagar, Daulath Singh, Alexy Glazyrin, Jill Moormeier

**Affiliations:** ^1^Internal Medicine, University of Missouri-Kansas City School of Medicine, 2301 Holmes Street, Kansas City, MO 64108, USA; ^2^Department of Pathology, University of Missouri-Kansas City School of Medicine, 2301 Holmes Street, Kansas City, MO 64108, USA; ^3^Department of Medicine, Hematology/Oncology, University of Missouri-Kansas City School of Medicine, 2301 Holmes Street, Kansas City, MO 64108, USA

## Abstract

Therapy related acute myelogenous leukemia (AML) and myelodysplastic syndromes (MDS) have been classically linked to alkylating agents and topoisomerase inhibitors. They constitute about 1% of all AMLs. There is less evidence on association of taxanes (paclitaxel and docetaxel) with these myeloid neoplasms. We present a case of paclitaxel therapy related acute myelogenous leukemia after treatment of endometrial cancer with a regimen containing paclitaxel and carboplatin. A 63-year-old female underwent surgery followed by a total of 6 cycles of chemotherapy with carboplatin and paclitaxel. Six months after last cycle of chemotherapy, she was diagnosed with myelodysplastic syndrome with refractory anemia and excess blasts. Six weeks later, she had worsening anemia and thrombocytopenia which prompted a bone marrow biopsy which revealed acute myelomonocytic leukemia. A thorough literature review revealed 12 other case reports where taxanes have been implicated in the development of therapy related myeloid neoplasm. Based on the timeline of events in our patient, paclitaxel is the likely culprit in the pathogenesis of this myeloid neoplasm. This rare but significantly grave adverse effect should be kept in consideration when deciding on treatment options for gynecological malignancies.

## 1. Introduction

Advancements in the treatment of gynecologic cancers with newer chemoradiotherapies have significantly improved the mortality and life expectancy of patients with these cancers. But with increased use of these chemotherapeutic agents, prescribers should be mindful of the potential long term side effects of these agents. One of the most serious complications of chemotherapeutic agents is dysplasia and malignancy.

Therapy related AML (t-AML) makes up about 10% of AML and has been studied and well documented in association with alkylating agents and topoisomerase inhibitors [1]. It usually involves a rapidly progressive disease, complex karyotype abnormalities, and poor prognosis with lesser response to treatment than archetypal AMLs.

Taxanes are the cornerstone of management of gynecological malignancies but recent data has linked them to cases of therapy related AML and MDS.

## 2. Case Presentation

We report a case of a 63-year-old Caucasian female found to have high grade endometrial carcinoma while undergoing evaluation for postmenopausal bleeding. The initial suspicion was from an abnormal pap smear done 3 years ago showing possible adenocarcinoma cells. Endometrial biopsy was done and showed high grade endometrial adenocarcinoma with serous features. The patient underwent surgical staging 4 months later with total abdominal hysterectomy, bilateral salpingo-oophorectomy, pelvic lymphadenectomy, and omentectomy. Out of the total 21 resected lymph nodes, one was involved with cancer and the carcinoma was staged at T3aN1M0, Stage 3.

Following surgical staging and tumor resection, the patient underwent chemotherapy with three cycles of carboplatin and paclitaxel at 21-day intervals, followed by pelvic radiotherapy (planned total dose 5040 cGy), and finally three additional cycles of carboplatin and paclitaxel. Initial doses of carboplatin were targeted at 750 mg/cycle (AUC = 6) and paclitaxel at 175 mg/m^2^. Because of extended duration radiation related Grade 3 colitis, radiation therapy was stopped after receipt of 1980 cGy. Patient then underwent 3 more cycles (cycles 4–6) of chemotherapy with carboplatin and paclitaxel. The total cumulative dose of paclitaxel was 1511 mg and that of carboplatin was 4312 mg.

Prior to beginning chemotherapy, the patient had normal blood counts except for a mild iron deficiency anemia (hemoglobin 11.2 gm/dL, white blood cell count 7200/mm^3^, and platelets 168,000/mm^3^). Her platelet count after completion of radiation therapy was 120,000–140,000/mm^3^, with mild anemia and a normal white blood cell count. After receiving the sixth and final dose of chemotherapy, her platelet count fell to 80,000/mm^3^ and did not recover.

**Figure 1:**
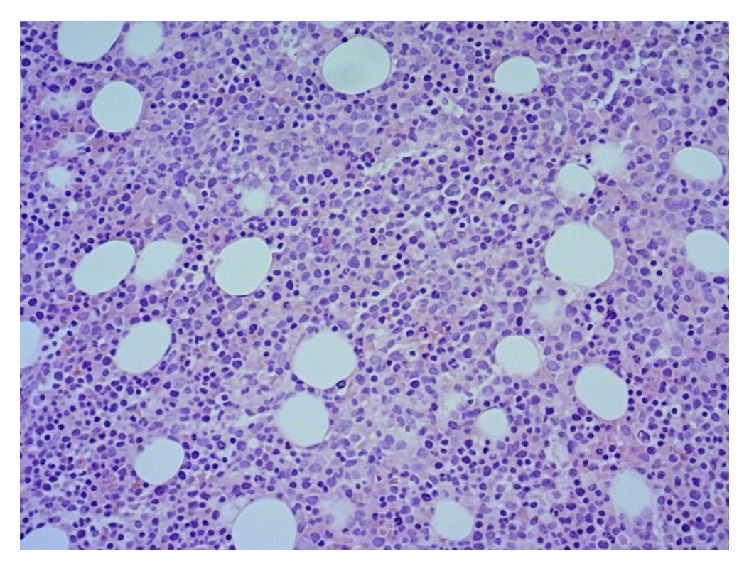
Bone marrow aspiration cytology (H&E stain, magnification 400x) shows hypercellular bone marrow with proliferating blasts replacing most of the normal bone marrow.

**Figure 2 fig2:**
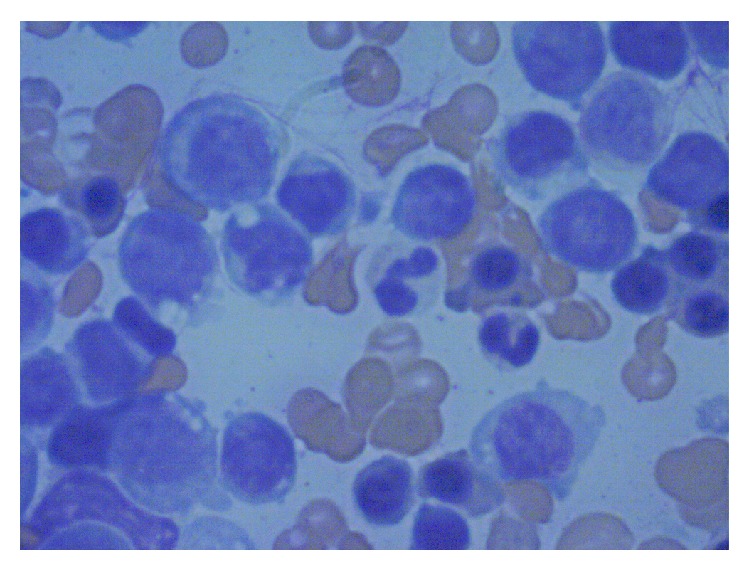
Bone marrow aspiration cytology (W&G stain, magnification 1000x) shows medium to large blast cells replacing normal bone marrow cellularity. Dysplastic granules and occasional vacuoles are observed.

**Figure 3:**
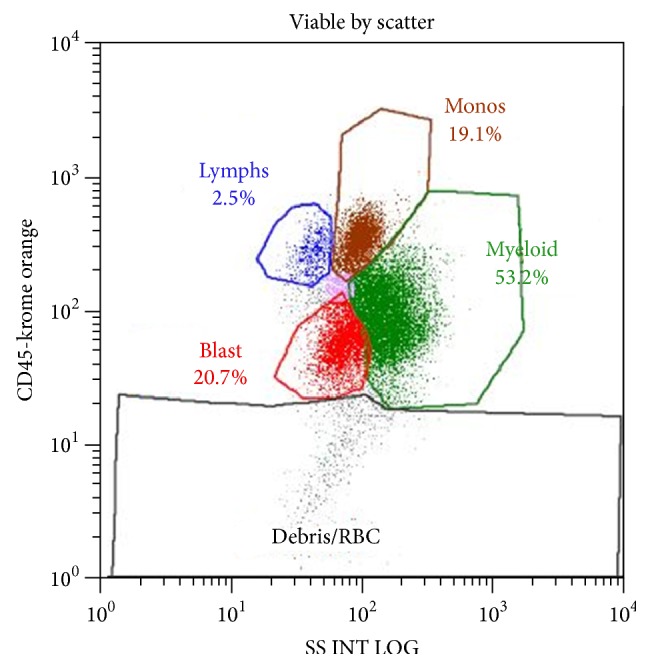
Bone marrow aspirate flow cytometry showing increased number of blasts and left shifted granulopoiesis.

**Figure 4:**
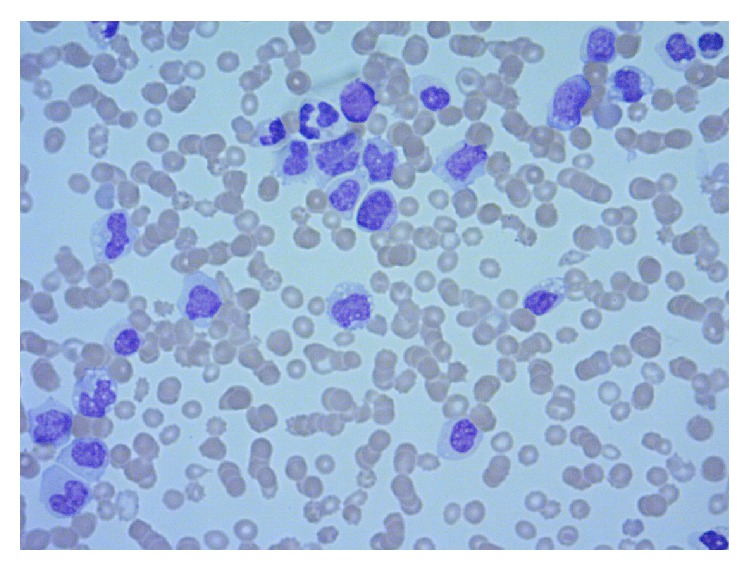
Peripheral blood smear: (W&G stain) show circulating blasts, dysplastic neutrophils, and nucleated red blood cells.

Six months after last cycle of chemotherapy, patient was hospitalized thrice over a period of two months with high grade fevers. She had worsening anemia and thrombocytopenia during this period which prompted a bone marrow biopsy. This revealed a hypercellular marrow with overall 5% myeloblasts with megaloblastic changes in the red cells and myeloid series and normal appearing megakaryocytes (Figures 1 [Fig fig2]–3). The peripheral blood showed marked anisocytosis with moderate normocytic normochromic anemia and left shifted myeloid series and thrombocytopenia (Figure 4). The findings were consistent with treatment related myelodysplastic syndrome with refractory anemia and excess blasts. Karyotyping revealed abnormal female karyotype: 45,XX,add(3)(p13),del(3)(q23q25),−5,add(5)(q13),add(7)(q11.2),der(17)t(5,17)(q15;q25).

Within six weeks of the diagnosis of myelodysplasia, she had progressed to acute leukemia. Initial lab work revealed leukocytosis up to 76000 with 22% blasts; anemia with Hb 6.4 g/dL; and thrombocytopenia with counts of 15,000/mm^3^. She was given a provisional diagnosis of therapy related AML. A bone marrow biopsy revealed hypercellular marrow with 29% of cells blasts. The blasts exhibited both myeloid and monocytic features and a diagnosis of acute myelomonocytic leukemia (AML; FAB classification: M4) was made. Peripheral smear also showed persistent leukocytosis with blasts >27% along with anemia and thrombocytopenia which eventually required transfusions.

Patient was offered treatment options including chemotherapy for AML and then possible stem cell transplant but decided not to pursue chemotherapy and opted for supportive treatment. She died a week later due to pneumonia and respiratory failure.

## 3. Discussion

Therapy related myeloid neoplasm (t-MN) is the term recently proposed by the World Health Organization to cover the spectrum of malignant disorders that were previously described as therapy related myelodysplastic syndromes (t-MDSs) or acute myeloid leukemia (t-AML).

Several chemotherapeutic regimens have been implicated in causing therapy related myeloid neoplasms. The most common of these agents are alkylating agents like cyclophosphamide, nitrosourea, or melphalan and topoisomerase inhibitors such as etoposide, daunorubicin, and doxorubicin. The latency period between first exposure to a cytotoxic agent and the development of therapy related myeloid neoplasms (t-MN) usually ranges from one to 10 years. This latency period and predominant cytogenetics observed often vary according to the chemotherapeutic agent used.

t-MNs after exposure to alkylating agents typically present after a latency period of approximately five to seven years [2]. Two-thirds of these patients are first recognized by evidence of myelodysplasia (usually trilineage dysplasia), marrow failure, and pancytopenia. The chromosomal abnormalities seen in these t-MNs often involve complex abnormalities and monosomies such as −5 or −7 that have been associated with unfavorable risk [3]. Combination of radiotherapy and chemotherapy was further shown to have increased risk of t-AML in some studies [4].

t-MNs that develop after the use of topoisomerase II inhibitors have a considerably shorter latency period of one to three years and most often present with overt leukemia and rarely with MDS or MDS/MPN [5]. The cytogenetic alterations typically apparent in these t-MNs often involve 11q23 abnormalities, such as t(9;11), or 21q22 abnormalities, such as t(8;21) or t(3;21) [6].

The platinum containing derivatives like carboplatin and cisplatin also have some leukemogenic potential. In a case control study by Travis et al., platinum containing agents were associated with an increased risk of* t*-MDS/*t*-AML [7]. Most of the cases seen in our literature review were treated concomitantly with taxanes (paclitaxel) and carboplatin. However, as reported in the study by Travis et al., carboplatin related t-AML have a higher latency period, averaging at 4 years (the minimum latency being 1.3 years in the report), which was not the case with our patient, making the platinum agent a less likely culprit.

Review of the English language literature revealed 12 other similar case reports where taxanes had been implicated in development of therapy related myelogenous leukemia (Table 1) [8–17]. Of these, mean time of onset of disease from beginning of chemotherapy and diagnosis of leukemia was about 8.75 months. The total dosage of paclitaxel used was specified clearly in 8 studies. Among these studies, the total mean dosage of paclitaxel used was 1342.5 mg which is the usual dose for gynecological malignancies, 175 mg/m^2^ [18, 19]. The malignant potential thus does not seem to be related to the dose of paclitaxel but rather to an idiosyncratic response to the treatment. It is unclear why taxanes lead to therapy related AML but treatment specific DNA damage is likely to be important in the pathogenesis [20]. Five of the nine cases which report the AML subtype revealed a M4 AML subtype (FAB classification). On review of the cytogenetics, there was no specific chromosomal abnormality that was common to the cases but almost all cases had a multitude of cytogenetic abnormalities which, not surprisingly, points to the poor prognosis seen in these cases. Larson, RA, in a recent review noted that it is the cytogenetics, and not merely prior exposures, which are the strongest predictors for outcome for these treatment related AMLs [21].

As seen in the literature review, the myelodysplastic syndromes and myelogenous malignancies associated with these agents are characterized by multiple cytogenetic abnormalities and typically have a very poor prognosis. Of the four patients who received treatment for secondary leukemia, two died within a year, one died 13 months later due to progression of the primary tumor, and one survived. Of the patients who did not receive treatment, the average survival was 7.5 months. In all of the reviewed cases, only two cases had gone into remission: the first described by Christodoulou et al. was detected early in the MDS stage and was treated two months after diagnosis with bone marrow transplantation [15] and the second case described by Seymour et al. had progressed to AML M4 subtype and had leptomeningeal leukemic infiltrate but was treated with intensified cytarabine therapy leading to remission [16]. However, due to the rarity of this adverse event, our review was limited by the paucity of similar case reports. Given that taxanes are commonly used in gynecological cancers, this rare but significant risk of myeloid neoplasms secondary to these agents should be taken into account while discussing and deciding on treatment options.

## Figures and Tables

**Table 1:** Case reports of taxane chemotherapy related myelogenous leukemias.

Author	Year	Age	Primary tumor	Docetaxel	Paclitaxel (total dose in mg)	Carboplatin (total dose in mg)	Karyotype	Latency of leukemia (period from initial treatment to onset of AML)	MDS	AML	Prognostic: time from Dx of AML to death
Ishikawa et al. [8]	2014	65	Ovarian cancer		1968	6480	45,XX,der(6)t(6:17)(p23;q21)inv(6)(p23q13),17, add(19)(p13)[4]/44,sl-5[10]/46,XX[5]	3 years 10 months	MDS-RCMD	—	15 m

Vanajakshi et al. [9]	2014	69	Ovarian cancer		1200	2700	53XX; trisomies 8, 12, 16, 18, 19, and +2mar JAK2V617F; 5q31 and 7q31 deletions	33 months	MDS-U	M4	1 m of AML

See et al. [10]	2006	54	Ovarian cancer		1710	6820	Abnormal karyotype with multiple complex abnormalities, including 45XX t(1,5)(q25;q35),del(4) (q21q26),ins(7)(p15;?),del(12)(p12),213,217,217, add(19)(p13.3), add(22)(q13),12mar in 11 metaphases and 43–45,XX,t(1;5) (q25;q35),del(4)(q21q26),ins(7)(p15;?) del(12)(p12),213,217,217,add(19)(p13.3), add(22)(q13),1224mar in 8 metaphases were identified at diagnosis of erythroleukemia	36 months	—	M6	10 m after dx of AML

Yeasmin et al. [11]	2008	73	Ovarian cancer		2085	1910	46,XX,der(5,19)(p10:q10),−6,del(7)(q?), add(12)(p11.2),−13,−17,+19	24 months	MDS-RAEB	M7	2 m

Kim et al. [12]	2006	64	NSCLC		525	1123	NR	27 months	MDS-CMML		9 m

Gupta et al. [13]	2014	64	Breast cancer		(Dose not specified)		46,XX,t(8;16)(p11.2;p13.3),der(22)t(1;22)(q21;q13)	2 years		M4	1 m

Melichar et al. [14]	2012	54	Ovarian and breast cancer		(Dose not specified)	Cisplatin equivalents 3798 mg	NR	5 years	MDS-RAEB	—	3 m

Melichar et al. [14]	2012	66	Ovarian cancer		31 cycles × 175 mg/m^2^	Cisplatin equivalents 4050 mg	NR	43 months		Unclassified	1 m

Christodoulou et al. [15]	2004	21	Mixed germ cell tumor (seminoma)		1000		47,XY,i(5)(p10),del(12)(p11),+i(12)(p10) [18]	10 months	MDS (RARS)	—	Survived

Seymour et al. [16]	1999	59	Ovarian cancer		1800	3600	NR	22 months		M4	Survived

Seymour et al. [16]	1999	54-55	Neuroendocrine cancer of ovary		450	750	NR	8 months		M4	10 weeks

Griesinger et al. [17]	2004	74	NSCLC stage IIIB	4 cycles × 100 mg/m^2^		4800	NR	40 months	MDS-RA	M2	40 m

Current	2014	63	Endometrial cancer		1511	4312	45,XX,add(3)(p13),del(3)(q23q25),−5,add(5)(q13), add(7)(q11.2),der(17)t(5,17)(q15;q25)	7.5 months	MDS-RAEB	M4	2 weeks

## Abbreviations

MDS: Myelodysplastic syndrome
AML: Acute myelogenous leukemia
t-AML: Therapy related acute myelogenous leukemia
t-MN: Therapy related myeloid neoplasm
cGY: Centigrays.
